# Editorial: Sortilin and Sortilin Partners in Physiology and Pathologies

**DOI:** 10.3389/fphar.2019.00791

**Published:** 2019-07-11

**Authors:** Jean Mazella, Marc Borsotto, Catherine Heurteaux

**Affiliations:** ^1^INSB, UMR7275 Institut de pharmacologie moléculaire et cellulaire (IPMC), Valbonne, France; ^2^UMR7275 Institut de pharmacologie moléculaire et cellulaire (IPMC), Valbonne, Provence-Alpes-Côte-d’Azur, France

**Keywords:** sortilin, sortilin partners, protein interactions, receptors, channels

The discovery of sortilin at the end of the 1990s from three different laboratories using three different biochemical approaches already predicted the multifunctional roles of the protein (Lin et al., 1997; Petersen et al., 1997; Mazella et al., 1998). Sortilin has been shown to exert important functions, such as receptor, co-receptor, and sorting protein to plasma membrane and/or lysosomes. In addition, the modifications of its expression indicated that sortilin plays a crucial role in numerous pathophysiological functions and in the central and peripheral organs as well. The central role of sortilin has been observed in Alzheimer’s and Parkinson’s diseases, depression, and the regulation of lipids and glucose homeostasis whose dysfunctions lead to cardiovascular risks.

This research topic aimed to collect original research studies as well as review and perspective articles that provide advances and future directions in the physiology and pathologies regulated by sortilin and its associated proteins, receptors, and channels.

Requested articles or reviews were not restricted to central pathologies (frontotemporal dementia, Alzheimer’s and Parkinson’s diseases, depression, etc.) but also concerned peripheral pathologies (diabetes and cardiovascular diseases).

From this presentation, we edited six articles and reviews describing the role of sortilin and sortilin-like SorLA and SorCS2 in immune-related processes, depressive-like behaviors, and alcohol-seeking symptoms and glucose homeostasis.

Chronologically, the first article “Altered Trek-1 Function in Sortilin Deficient Mice Results in Decreased Depressive-Like Behavior” by Moreno et al. described the interesting observation that, in sortilin-deficient mice, the central function and the cellular location of the two-pore potassium channel TREK-1 are modified, leading mice to display a reduced depressive-like behavior. These observations 1) confirm initial data identifying TREK-1 as a potent target in depression (Heurteaux et al., 2006) and 2) are coherent with the involvement of sortilin to address TREK-1 channels at the plasma membrane of neurons (Mazella et al., 2010).

The review entitled “Regulatory Roles of Sortilin and SorLA in Immune-Related Processes” by Talbot et al. is a summary of the role of the two members of the Vps10p domain receptor family, sortilin and SorLA, in the modulation of processes related to the immune system. For example, SorLA has been shown to be involved in the regulation of interleukin-6 signaling as well as in monocyte migration. In contrast, several studies highlighted the important functions of sortilin in microglia by regulating proinflammatory cytokines as well as in phagosome fusion and pathogen clearance. This review introduced new peripheral functions of two proteins whose roles have been extensively studied in many human cerebral disorders such as Alzheimer’s and Parkinson’s diseases and depression.

The mini-review “The Involvement of Sortilin/NTSR3 in Depression as the Progenitor of Spadin and Its Role in the Membrane Expression of TREK-1” by Mazella et al. described how the potent antidepressant spadin has been discovered from the observation that both TREK-1 and sortilin-deficient mice behaved very similarly in several depressive-like behavioral tests. The review also summarized the importance of the complex TREK-1/sortilin for the function of the channel.

The mini-review “Sortilin in Glucose Homeostasis: From Accessory Protein to Key Player?” by Blondeau et al. described a new peripheral function of sortilin focusing on molecular mechanisms regulating blood glucose homeostasis and insulin signaling. The authors particularly discussed the role of the neurotensinergic system in these endocrine functions by the interaction of neurotensin receptors with sortilin that appears crucial for beta pancreatic cell survival.

The review “Role of TREK-1 in Health and Disease, Focus on the Central Nervous System” by Djillani et al. focused on the numerous studies on the physiological and pathophysiological roles of one of the sortilin partners, the potassium channel TREK-1, as its discovery by molecular cloning in 1996 (Fink et al., 1996) up to its recent important involvement in depression, post-stroke depression, and cardiac functions. The review also discussed the agonists and antagonists of the channel as potent pharmacological tools to modulate the important functions controlled by TREK-1.

Finally, the original research article “Reduced Alcohol Seeking and Withdrawal Symptoms in Mice Lacking the BDNF Receptor SorCS2” by Olsen et al. discussed the role of a member of the Vps10p domain receptor family, named SorCS2, which could be implicated in the repetitive and uncontrolled intake of alcohol, a fact that leads to a severe consequence for affected individuals as well as their families. Interestingly, they showed that SorCS2 could be responsible, at least in mice, for the alcohol preference and for the handling-induced seizures in response to alcohol withdrawal after extended alcohol exposure. These observations are likely due through the interaction of SorCS2 with the brain-derived neurotrophic factor signaling system by interacting with its two receptors, TrkB and p75NTR.

We would like to thank all authors for their participation in this research topic on sortilin and sortilin partners in pathologies in pharmacology. Although all the expected scientific fields have not been represented in this research topic, we hope that it becomes a major reference for those working in the field.

## Conflict of Interest Statement

The authors declare that the research was conducted in the absence of any commercial or financial relationships that could be construed as a potential conflict of interest.
